# Revealing Local Temporal Profile of Laser Pulses of Intensity above 10^14^ W/cm^2^

**DOI:** 10.3390/s23063101

**Published:** 2023-03-14

**Authors:** Qi Lu, Xiang Zhang, Arnaud Couairon, Yi Liu

**Affiliations:** 1 Shanghai Key Lab of Modern Optical System, University of Shanghai for Science and Technology, 516, Jungong Road, Shanghai 200093, China; 2 Centre de Physique Théorique, CNRS, Ecole Polytechnique, F-91128 Palaiseau, France; 3 CAS Center for Excellence in Ultra-Intense Laser Science, Shanghai 201800, China

**Keywords:** second harmonic, gas plasma, temporal profile

## Abstract

We demonstrated a method for in situ temporal characterization of an intense femtosecond laser pulse around its focus where the laser intensity exceeds 10^14^ W/cm^2^. Our method is based on the second harmonic generation (SHG) by a relatively weak femtosecond probe pulse and the intense femtosecond pulses under analysis in the gas plasma. With the increase in the gas pressure, it was found that the incident pulse evolves from a Gaussian profile to a more complicated structure featured by multiple peaks in the temporal domain. Numerical simulations of filamentation propagation support the experimental observations of temporal evolution. This simple method can be applied to many situations involving femtosecond laser–gas interaction, when the temporal profile of the femtosecond pump laser pulse with an intensity above 10^14^ W/cm^2^ cannot be measured in traditional ways.

## 1. Introduction

The temporal characterization of femtosecond laser pulses is basic and important for the development of ultrafast laser technology and the study of the nonlinear interaction of ultrafast lasers with materials [1]. Up to now, many kinds of techniques based on different principles have been developed, such as autocorrelator [2,3], Frequency Resolved Optical Gating (FROG) [4], Spectral Phase Interferometry for Direct Electric-field Reconstruction (SPIDER) [5,6], d-scan [7,8], etc. All the techniques sample the optical pulse under investigation with a pulse energy on the level of nanojoules to microjoules due to the limitation of optical damage of the optical components inside the measurement devices. On the other hand, the focused laser intensity can easily go beyond 10^14^ W/cm^2^, even with a commercial femtosecond laser system delivering millijoule-level pulses, and such high intensity can result in the ionization of any materials including gases. As a result, it is impossible to measure the temporal profile of such focused intense pulses locally with the above devices. Therefore, the temporal profile of the laser pulse and its evolution during laser–gas interaction around the beam focus largely remains out of reach since traditional detectors or devices will be damaged by the intense pulses. Recently, for intense few-cycle pulses (10 fs) around its focus, it has been demonstrated that the stereographic above-threshold ionization (ATI) measurement of photoelectrons provides a pulse length measurement at the position of the laser–gas interaction [9]. This method is based on the fact that the ATI spectrum of noble gases such as Xe is dependent upon the carrier envelope phase (CEP) and that this dependence increases as the pulse length decreases. Nevertheless, this method is applicable only for a few cycle pulses and sophisticated apparatus for electron detection is necessary. In most cases, information about the temporal profile of the pulses around the focus during interaction can only be assessed by numerical simulations [10,11]. Therefore, direct characterization of the pulse temporal profile at the location of the laser–gas interaction where photoionization occurs is highly desired.

In this study, we proposed and demonstrated a nonlinear optical method for in situ characterization of the temporal profile of the intense femtosecond laser pulse during the laser–gas interaction around its focus. We proposed that the second harmonic generation in the inhomogeneous gas plasma by a weak femtosecond probe pulse together with the intense pump pulses under test provide a second-order cross-correlation of the two pulses. In the experiments, we recorded the SHG signal from nitrogen, air, or argon plasma as a function of gas pressure and observed the temporal evolution of the intense pulse from a Gaussian profile to a multiple-peak structure for higher gas pressure. Our observations were confirmed by numerical simulation of nonlinear pulse propagation where multiple peaks in the temporal domain are produced due to a competition between Kerr self-focusing and plasma defocusing. We believe that this simple method can find applications for pulse characterization in many situations of laser–gas interactions such as high-order harmonic generation, laser particle acceleration, generation of Terahertz radiation from laser-induced plasma, etc.

## 2. Materials and Methods

The origin of the proposed method stems from the fact that a second harmonic signal from the laser-induced air plasma has been observed by several groups [12,13,14]. Although air is a centrosymmetric media that prohibits the generation of even order harmonics in the perturbative regime, the second harmonic generation (SHG) of the fundamental 800 nm pulses has been widely observed in gas plasma [12,13,14]. The main mechanism underlying this SHG has been largely attributed to the gradient of photoionization-induced plasma caused by the ponderomotive force [12,13,14]. It has been observed that the SHG presents a nearly quadratic dependence on the laser intensity above the ionization threshold intensity [12,13]. Taking advantage of this quadratic dependence, we conceive that this SHG in the plasma can be used for cross-correlation of an unknown laser field with a reference pulse, such as the traditional cross-correlation based on the second harmonic generation with a Beta-Barium Borate (BBO) crystal.

**Figure 1:**
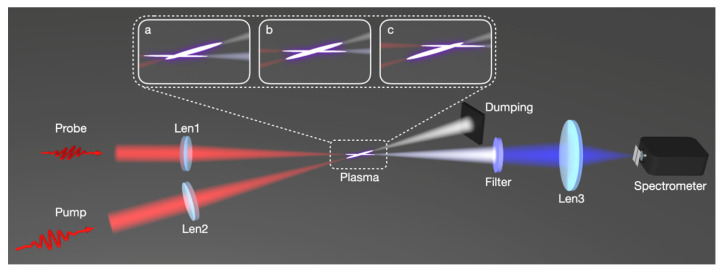
Schematic diagram of experimental setup. Illustration a, b, c shows the pump pulse at different locations along the plasma string was detected by the probe pulse.

Femtosecond laser pulses at a central wavelength of 800 nm from a Ti: sapphire amplifier (Coherent Legend DUO, Coherent Inc, Santa Clara, CA, USA) were used in the experiments. The duration of the pulses was 35 fs and the maximum pulse energy was 12 mJ. The main experimental setup is depicted in Figure 1. In Figure 1, the pulses were focused by a focal lens of f = 30 cm in a gas chamber and the generated SH in the forward direction were detected after a dichroic mirror (high reflectivity at 800 nm and high transmission at 400 nm) and proper glass filters (BG 39 and BG 40). With this setup, we measured the dependence of the SHG intensity on the pump laser intensity. In the second experiment, the femtosecond pulses were divided into two beams. The relatively stronger 800 nm laser beam with the pump energy of ~3.4 mJ served as the pump pulse to produce a filamentary plasma string in the gas chamber. Another weak 800 nm laser beam was used as a probe pulse. Both the pump and probe laser pulses were focused by a fused silica lens of f = 30 cm into the gas chamber. The two beams cross each other at a crossing angle of ~10°. This angle cannot be smaller due to the scattered light from the pump beam into the spectrometer installed downstream of the probe beam. The focal lens of the pump pulse is installed on a three-dimensional translation stage so that the different locations of the plasma string produced by the pump pulse can be spatially overlapped with the focus of the probe pulse. In the experiment, we concentrated on the second harmonic signal generated in the probe beam path. After passing through proper glass filters (BG 39 and BG 40) and bandpass filters with a central wavelength at 400 nm, the spectrum of the second harmonic in the probe beam path was recorded by a spectrometer. The gas pressure and specie can be changed inside the chamber.

## 3. Results

**Figure 2:**
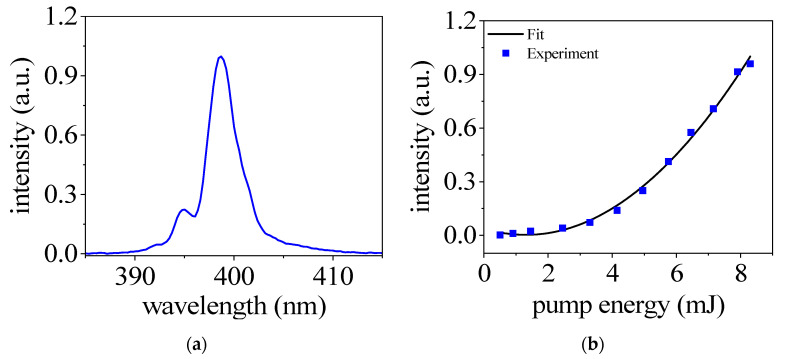
(**a**) Spectra of second harmonic generated in nitrogen with pump energy of 8.7 mJ. (**b**) The intensity of the SH signal as a function of the pump energy. The gas pressure is at 20 mbar in both (**a**,**b**).

In Figure 2a, we present the spectra of the second harmonic generated in 20 mbar nitrogen gas with a pump energy of 8.7 mJ. It can be seen that the spectral range of the second harmonic is from 390 nm to 410 nm, and the center is about 398 nm. We also measured the intensity of the second harmonic signal as a function of the pump laser energy, shown in Figure 2b. The nitrogen gas pressure is 20 mbar. The signal shows a good agreement with a quadratic fit, indicating that the SHG inside the plasma can be used as a second-order nonlinear optical process.

With the scaling law of the SH signal on the pump pulse intensity confirmed, we can perform cross-correlation experiments of an intense femtosecond laser pulse under investigation with a relatively weak probe pulse. To separate the second harmonic signal due to the cross-correlation of the two pulses from that generated by the intense pulse itself, the probe pulse intersects the intense pulse with an angle of ~10° in our experiments. At a distance ~50 cm away from the plasma, the beam spot of the probe is well separated from that of the intense pulse. The second harmonic signal produced due to the interaction of the two pulses was then recorded as a function of the delay *τ* between them. In the experiment, shortwave pass filter (BG39) and interference filter centered around 400 nm were used to isolate the second harmonic radiation from the residual 800 nm probe pulse.

**Figure 3:**
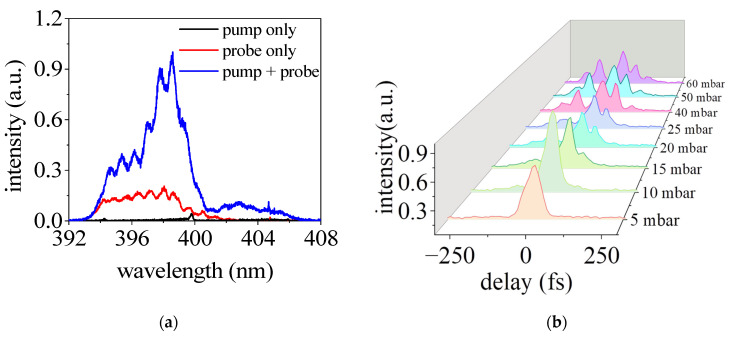
(**a**) SH spectra generated in the cases with pump and without pump pulse. (**b**) The signal intensities of SH at around 398 nm as a function of the time delay between pump and probe pulses with different nitrogen pressure.

Figure 3a shows the spectrum of second harmonic collected at the end of the probe beam path. The time delay between the pump and probe pulses *τ* was set to be zero. For comparison, the spectrum measured with only the pump (blue line) or probe pulses (red line) are also shown. In the case of only pump pulses, a very weak second harmonic signal can be observed, which is due to the scattered light from the pump beam path to the detector situated on the propagation axis of the probe beam. This residual SH signal can be eliminated with a larger angle between the pump and probe beams. With only the probe pulse, the SH spectrum shows a relatively wide range from 393 nm to 402 nm. With the presence of both the pump and probe pulses, the intensity of SH is significantly enhanced and the spectrum extends towards the longwave length side. This enhanced second harmonic signal corresponds to the cross-correlation signal and can be exploited for the temporal characterization of an unknown pulse.

To demonstrate the characterization of the temporal profile of an intense femtosecond laser pulse at focus, we measured the SH signal intensity as a function of the time delay *τ* for different gas pressures. The results are presented in Figure 3b. The zero time delay is set at the instant when the signal intensity is the strongest, and the positive time delay corresponds to the fact that the probe pulse lags behind the pump pulse. At relatively low pressures of 5 and 10 mbar, we observed only one peak of the second harmonic signal in the temporal domain. This agrees with the expectation that the pulse experiences linear propagation in very low gas pressure and the temporal profile remains the initial Gaussian profile. In the linear pulse propagation regime, the laser intensity can be estimated to be 2.6 × 10^16^ W/cm^2^ around the focus by consideration of a focus diameter of 22 μm. With the increase in the gas pressure, it was found that the incident pulse evolves into a more complicated structure featured by two or more peaks in the temporal domain. Similar results to Figure 3 have also been obtained in argon gas and air for increasing pressures. It has been well established that in the filamentation regime the laser intensity is clamped above 1.5 × 10^14^ W/cm^2^ for a millijoule level pulse [15]. Therefore, this method provided a simple in situ temporal characterization of the intense pulses with intensity above 10^14^ W/cm^2^ and revealed the rich temporal evolution of the pulse during nonlinear propagation. This pulse splitting and temporal transformation of the pulse will be elaborated later with numerical simulations.

**Figure 4:**
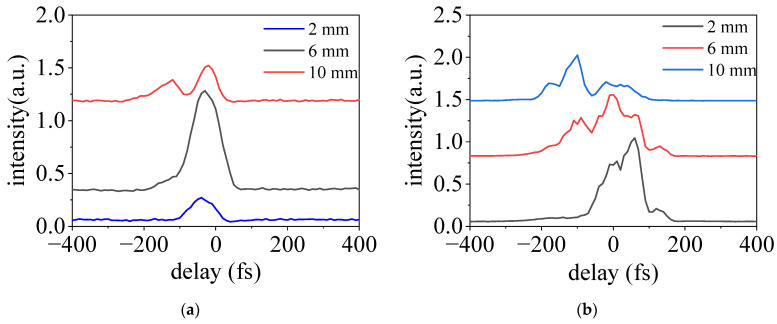
The temporal evolution of the SH signal generated in the nitrogen gas at the pressure of 15 mbar (**a**) and 80 mbar (**b**); 2 mm, 6 mm, and 10 mm correspond to the front, middle, and rear sections of the plasma, respectively.

To obtain insight into the temporal evolution of the incident pulse along the filamentary plasma, we further measured the SH intensity as a function of time delay *τ* at a different location of the plasma. The visible filament was about 10 mm long in this experiment. The results are presented in Figure 4. Here, 2 mm, 6 mm, and 10 mm correspond to the front, middle, and rear parts of the plasma, respectively. As shown in Figure 4a, in a relatively lower pressure of 15 mbar, we can observe that the temporal profile of the SH signal is a Gaussian profile at 2 mm and 6 mm, while two peaks appear in the time domain at 10 mm. For a relatively higher pressure of 80 mbar, the temporal profile of the SH signal starts to show two or more peaks at the position of 2 mm and 6 mm, as illustrated in Figure 4b.

## 4. Discussion

To confirm our above observation of the temporal transformation dynamic of the pulse during nonlinear propagation, we performed a numerical simulation of the nonlinear pulse propagation in argon gas. We propagate a Gaussian pulse with experimental parameters, by means of numerical simulations of the Unidirectional Pulse Propagation Equation (UPPE) [16,17], coupled with the matter response,

(1) $$\frac{\partial \hat{E}}{\partial z}=ik_{z}\hat{E}+i\frac{\mu_{0}\omega }{2k_{z}}\left[\omega {\hat{P}}_{nl}+i\left({\hat{J}}_{f}+{\hat{J}}_{a}\right)\right]$$

where $\hat{E}(k_{x},k_{y,}\;\omega ,z$) denotes the Fourier component of the angularly resolved frequency spectrum of the laser pulse, $k_{z}\left(k_{x},k_{y,}\;\omega \right)\equiv {\left[k^{2}\left(\omega \right)-k_{x}^{2}-k_{y}^{2}\right]}^{1/2}$ is the propagation constant with $k_{x}$, $k_{y}$, and ω being the spatial and temporal angular frequencies, and *μ*_0_ is the vacuum permeability. Dispersion or argon is taken into account via the frequency-dependent refractive index, *n*(*ω*), given by the Sellmeier relation in [18], which appears in the propagation constant *k*(*ω*) = *n*(*ω*)*ω/c*, with *c*, the speed of light in vacuum. The nonlinear terms in (1) include the nonlinear polarization $P_{nl}$ induced by bound electrons, the current density induced by free electrons $J_{f}$, and a phenomenological current density $J_{a}$ accounting for nonlinear absorption. They are all calculated in the space-time domain, and their angularly resolved frequency spectra are obtained by Fourier transforms at each propagation step for space marching the spectral components of the axially symmetric field *E*(*z, r, t*) using (1). The nonlinear polarization describes the optical Kerr effect,

(2) $$P_{nl}\left(z,r,t\right)=\epsilon_{0}\chi^{3}E^{3}\left(z,r,t\right)$$

where $\epsilon_{0}$ is the vacuum permittivity, $\chi^{3}=4\epsilon_{0}cn_{0}^{2}n_{2}/3$ is the cubic susceptibility, with *n*_2_ being the nonlinear index and *n*_0_ being the medium refractive index at the pulse central frequency $\omega_{0}$. The free electron current is calculated from a frequency-resolved Drude model that describes the excitation of the current of free electrons $J_{f}\left(z,r,t\right)$ by the propagating electric field $E\left(z,r,t\right)$, coupled with the photoionization model describing the generation of free electrons of density $\rho_{e}\left(z,\;r,\;t\right)$, at the rate $W\left(\left|E\right|\right)$,

(3) $$\frac{\partial J_{f}}{\partial t}+\nu_{c}J_{f}=\frac{q_{e}^{2}}{m_{e}}\rho_{e}\left(t\right)E\left(t\right)$$

(4) $$\frac{\partial \rho_{e}}{\partial t}=W\left(\left|E\right|\right)\left(\rho_{nt}-\rho_{e}\left(t\right)\right)$$

Here, $q_{e}$ and $m_{e}$ are the charge and mass of the electron, $\nu_{c}=$ 5.26 THz bar^−1^ is the collision frequency [19], and $\rho_{nt}=2.5\times 10^{19}$ cm^−3^ bar^−1^ is the density of neutral argon atoms. The current that is responsible for nonlinear absorption reads

(5) $$J_{a}=\epsilon_{0}cn_{0}U_{i}\frac{\partial \rho_{e}}{\partial t}\frac{E}{\rho_{nt}|E{|}^{2}}$$

where $U_{i}=15.76$ eV is the ionization potential of argon. 

Photoionization rates $W\left(\left|E\right|\right)$ are calculated from a Keldysh-like formulation [20], including further developments by PPT [21] and Shcheblanov et al. [22]. The value of the nonlinear index coefficient for argon is $n_{2}=10\times 10^{-20}$ cm^−2^ W^−1^ bar^−1^. Changing the pressure of argon results in a modification of the index of refraction according to the Lorentz–Lorenz equation. The index of refraction is then dependent on the pressure *p* through the following relations

(6) $$\chi =\frac{n_{0}^{2}-1}{n_{0}^{2}+2},n\left(p\right)={\left(\frac{1+2p\chi }{1-p\chi }\right)}^{\frac{1}{2}}$$

where $n_{0}$ denotes the frequency dependent index of refraction at 1 bar, and *p* is expressed in bar. Other model parameters are pressure dependent. For instance, the nonlinear index coefficient, the density of neutral atoms, and the collision frequency are multiplied by the pressure.

The simulations are performed for carrier-wave-resolved fields in axially symmetrical geometry (*t*; *r*) + *z* with a temporal domain of 2.4 ps, using 16,384 evenly distributed grid points, leading to a temporal resolution of 0.14 fs (18 grid points per wavelength). In the transverse domain r, the initial beam has the largest radius of 5.5 mm. We choose a 10 mm large r-domain with 600 unevenly distributed grid points giving the best resolution of 12 μm in the region close to the axis, increasing to the edge of the domain. Space marching is performed with adaptative z-step as required to resolve nonlinearity within the focal region. The numerical grid is 2^12^ (*t*) × 600 (*r*), and a single z-step needs to be kept in computer memory for space marching, so our solver requires 1.2 GB RAM. Standard tests to benchmark propagation codes are presented in [16] and were successfully passed.

**Figure 5:**
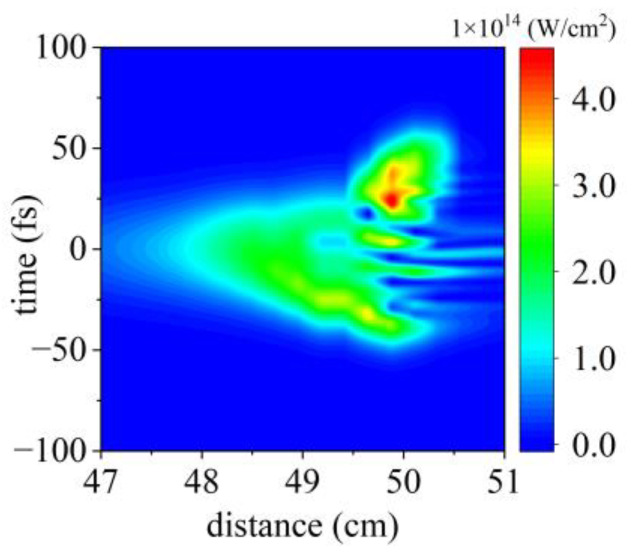
The on-axis laser intensity as a function of the propagation distance for a 40 fs, 3.4 mJ pulse. The gas pressure is 200 mbar.

For the initial conditions for simulation, we considered a 40 fs pulse at the central wavelength of 800 nm. The pulse energy is 3.4 mJ. The initial beam with a width of 11 mm is focused by a lens of *f* = 50 cm in nitrogen gas of pressure between 5 and 200 mbar. In Figure 5, we present the temporal profile of the on-axis intensity as a function of propagation distance. Around the focus, the initial Gaussian temporal profile gradually evolves into a complex structure featured by multiple peaks due to the nonlinear interaction of the laser and the gaseous plasma.

**Figure 6:**
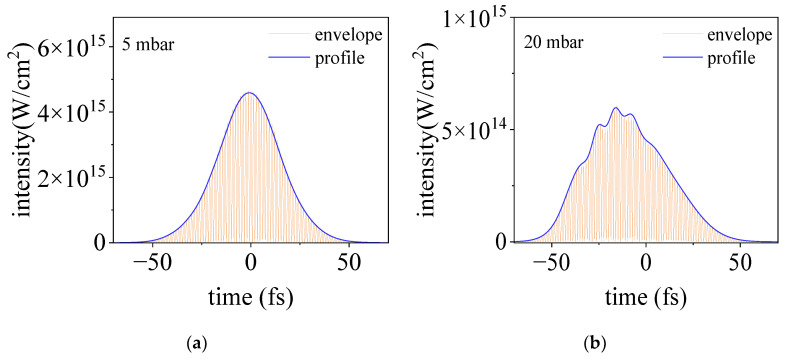

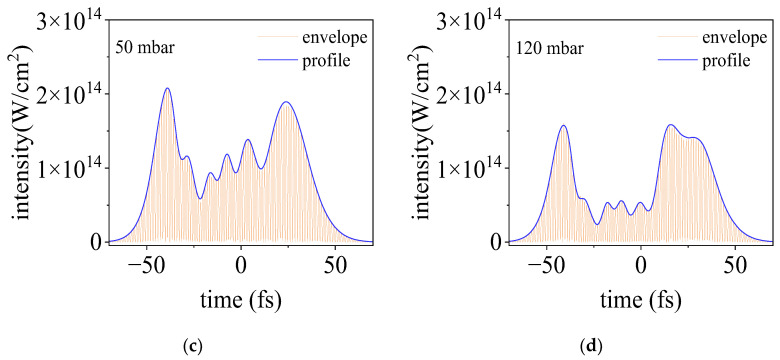
The temporal evolution of an intense femtosecond laser pulse propagating in argon. The gas pressures for (**a**–**d**) are 5, 20, 50, and 120 mbar, respectively.

In Figure 6, we presented the temporal profile of the laser intensity on the axis (*r* = 0) at the focus for different gas pressures. For a relatively low gas pressure of 5 mbar, the pulse propagates linearly and it keeps the initial temporal profile. For higher pressures, more and more complicated temporal structures appear, which agree qualitatively with our experimental observations and justify the principle of the proposed method. We would like to point out that the difference between the experimental observations and the simulated results can be due to the fact that in our experiments we used a probe pulse of 35 fs which is not short enough to detect the subtle temporal transformation of the pump pulse. We think that a more precise temporal characterization of an intense pulse can be obtained by the employment of a few-cycle probe pulse and the usage of deconvolution of the measured results with the temporal profile of the probe pulse.

## 5. Conclusions

In conclusion, we demonstrated an in situ nonlinear optical method to characterize the temporal profile of an intense femtosecond pulse around its focal zone where the laser intensity is on the order of 10^14^–10^16^ W/cm^2^. The method is based on the second harmonic generation in the gas plasma at the interception of the weak probe pulse and the intense pump pulse under measurement. We have shown that around the focus of the intense pulse where photoionization occurs, its temporal profile evolves from an initial Gaussian profile to a multiple-peak structure for increasing gas pressure. Tests in different gases including nitrogen, argon, and air confirmed the wide applicability of the method. Moreover, the evolution of the temporal profile of the pulse along the propagation direction was also demonstrated. Our experimental observations were confirmed by numerical simulation of the nonlinear pulse propagation. This method should be also applicable inside isotropic solids such as fused silica. We believe that this simple in situ method for temporal characterization of the ionizing laser pulse can find wide applications in the domain of high-field physics.

## Data Availability

The data supporting the findings of this study are available from the corresponding authors upon request.
